# Sacrococcygeal teratoma with preterm delivery: a case report

**DOI:** 10.1186/s13256-020-02395-9

**Published:** 2020-06-19

**Authors:** Anna Moreno Baró, Silvia Pina Perez, Montserrat Mestre Costa, Cristina Lesmes Heredia, Laura Serra Azuara, Judith Lleberia Juanos, Marc Zamora Lapiedra

**Affiliations:** Gynecology and Obstetrics Department, Corporació Sanitària Parc Taulí, Parc Taulí s/n, 08208 Sabadell, Spain

**Keywords:** Sacrococcygeal teratoma, Pregnancy, Obstetric ultrasound, Preterm birth

## Abstract

**Background:**

Sacrococcygeal teratoma is one of the most frequently prenatally diagnosed neoplasias. Obstetric ultrasound has a role in the diagnosis and management of these tumors during pregnancy. In this report, we describe a multidisciplinary approach in a case of a patient with sacrococcygeal teratomas and preterm delivery, as well as postnatal outcomes.

**Case presentation:**

A 26-year-old Caucasian woman at 20.3 weeks of gestation with a normal gestational course and no relevant medical or surgical history was referred to our institution with a sacrococcygeal mass diagnosis. Magnetic resonance imaging confirmed the diagnosis of sacrococcygeal teratoma type I according to the Altman classification. Follow-up with ultrasound showed an increase in the size of the mass up to 190 × 150 mm, high Doppler flow, and severe polyhydramnios. At 35.1 weeks of gestation, the patient had premature rupture of membranes, and an emergency cesarean section was performed due to recurrent late decelerations detected by fetal heart rate monitoring. Afterward, surgery was performed successfully at 36 hours of life. Posterior controls revealed normal and healthy child growth.

**Conclusions:**

This case report demonstrates the importance of a multidisciplinary approach to offer the best neonatal outcomes by performing early surgery, as well as the need for follow-up by ultrasound in order to minimize complications by assessing mass growth, Doppler flow, and amniotic fluid.

## Background

Sacrococcygeal teratomas (SCTs) are the most common extragonadal germ cell tumors (GCTs) in infants and young children, with an estimated incidence of 1 in 27,000 fetuses [1, 2]. This makes SCTs one of the most frequently diagnosed neoplasias prenatally.

In adulthood, GCTs are often located in other sites, such as the mediastinum and retroperitoneum, and the incidence of malignant elements is higher than in children [3].

SCTs are comprised of different types of tissues that come from at least two of the three germ cell layers, and, depending on the tissues that are included, they are divided into mature, immature, or malignant teratomas (which generally are not seen in infants) [4].

As shown in our patient’s case, obstetric ultrasound let us make a prenatal diagnosis, normally in the second trimester by routine sonography, and it is also an important tool in the evaluation and monitoring of the tumor over the course of gestation in order to identify fetuses at increased risk of complications and to plan a multidisciplinary treatment or intervention, when appropriate [5]. What our case brings to light is confirming that an appropriate approach to these tumors (even if it is as huge as in our patient’s case) with ultrasound monitoring and early surgery after birth results in good outcomes for both mother and infant.

## Case presentation

A 26-year-old Caucasian woman, gravida 2, para 1, with a spontaneous normal vaginal delivery 2 years ago was referred to our institution at 20.3 weeks of gestation after a sonographic finding of a sacrococcygeal mass of 26 × 24 mm in a male fetus.

The patient had no family history of birth defects or genetic disorders. She did not have any medical or surgical history, and she had no alcohol or smoking habit. She had no relationship with the father of her fetus and received no drug therapy while pregnant.

**Fig. 1:**
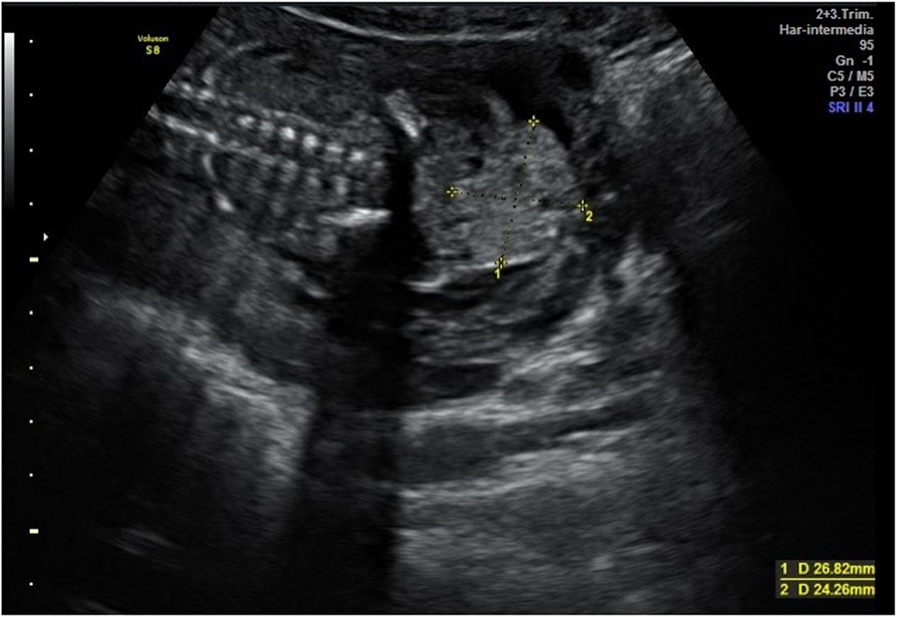
First Scan: exophytic mixed echogenic mass arising from the sacrococcygeal region

She had a normal gestational course with low risk of aneuploidies in the first-trimester screening and a normal first trimester scan at 13 weeks. Her sonographic examination revealed a single intrauterine pregnancy with an estimated gestational age of 20 weeks. The study revealed an exophytic, mixed echogenic mass arising from the sacrococcygeal region with high vascularization seen on Doppler flow (Fig. 1). The examination showed adequate amniotic fluid, and no other abnormalities were detected.

**Fig. 2:**
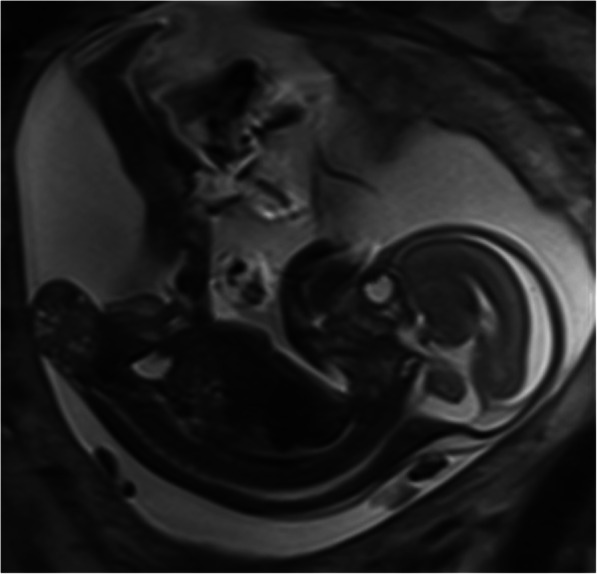
Magnetic Resonance Imaging (MRI) showing the sacrococcygeal teratoma, Type I and confirming the diagnosis

Magnetic resonance imaging was performed, which confirmed the diagnosis. There was no evidence of possible invasion of the fetal pelvis or abdomen. The spine appeared intact. The lower extremities, fetal kidneys, and bladder appeared normal. On the basis of these findings, a diagnosis of external variety, type I in the Altman classification, was confirmed (Fig. 2).

Amniocentesis guided by ultrasound scanning was done with normal karyotype and microarray results. The result of a fetal echocardiographic scan was normal.

**Fig. 3:**
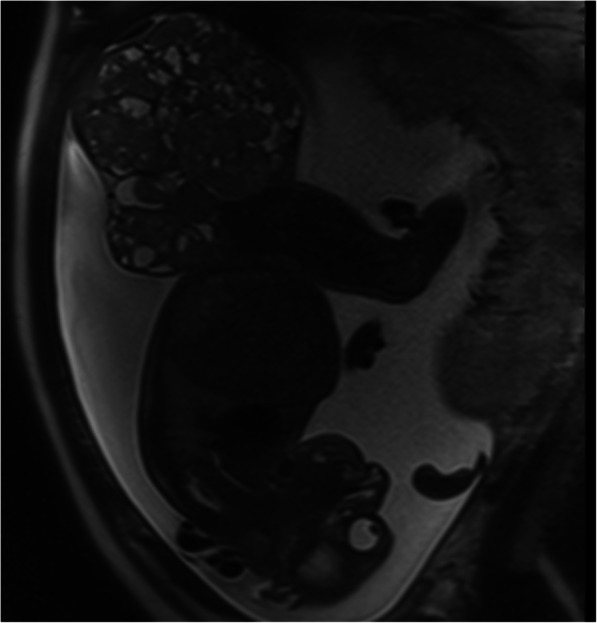
Follow-up MRI at 29 weeks where it’s seen the growth of the sacrococcygeal teratoma

**Fig. 4:**
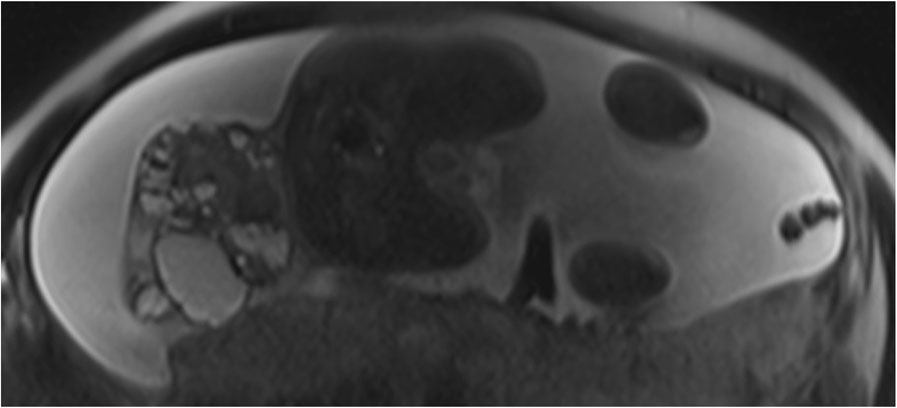
Same MRI, coronal cut of the mixed mass

**Fig. 5:**
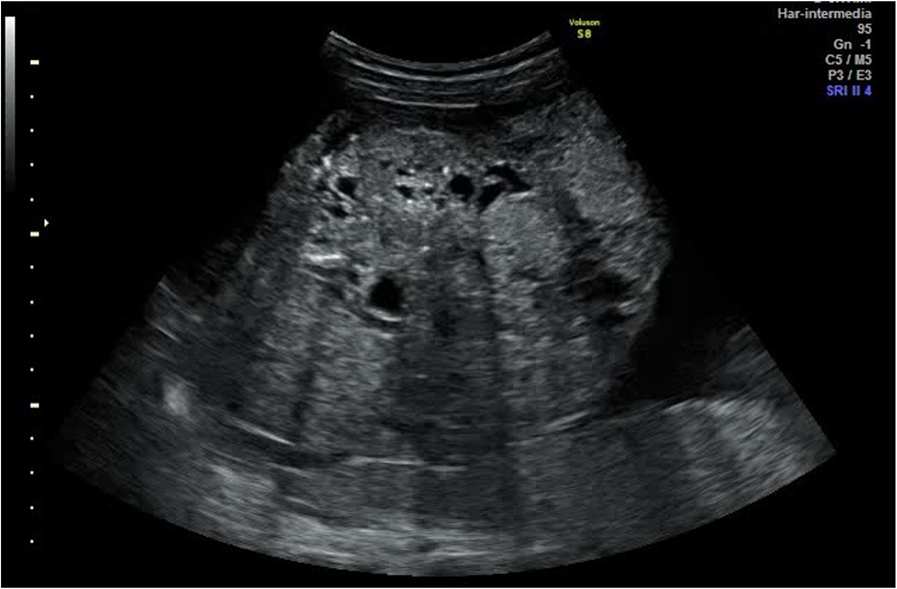
Follow- up scan at 31 weeks showing an increase where the teratoma is too large to be shown in a single image

The patient was scheduled for follow-up by ultrasound weekly (Figs. 3 and 4). These scans showed an increase in the size of the mass up to 190 × 150 mm with high Doppler flow and severe polyhydramnios (amniotic fluid index 37) (Fig. 5). The patient developed gestational diabetes, which required insulin treatment.

At 33.6 weeks of gestation, the patient was admitted to the obstetric ward for preterm labor. Her vital signs were normal (body temperature 36 °C, pulse rate 90 beats/minute, and blood pressure 135/82 mmHg), as was her physical examination. Treatment with a corticosteroid (12 mg intramuscularly, twice) and atosiban was started. The tocolysis was effective, and an elective cesarean section was scheduled at 35 weeks of gestation in conjunction with the neonatal service and the pediatric surgeon. However, 1 day before the scheduled cesarean section, the patient had premature rupture of membranes. Fetal heart rate monitoring revealed recurrent late decelerations at that moment, and an emergency lower segment cesarean section was performed.

**Fig. 6:**
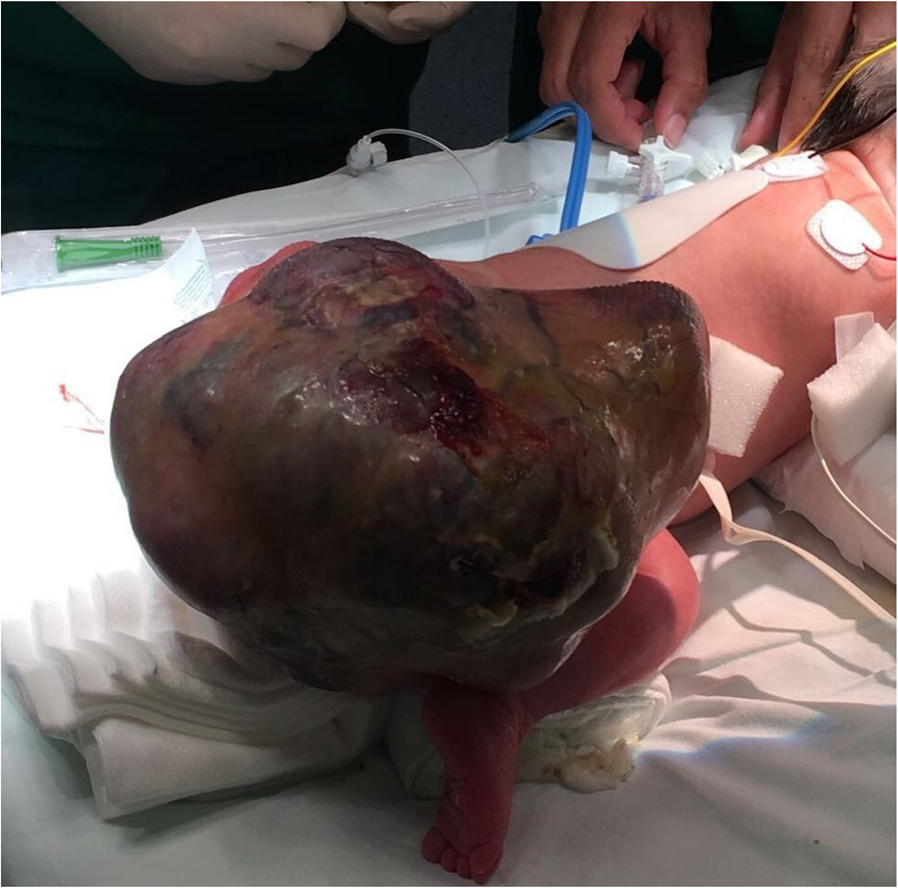
The newborn in posterior view with the presence of the sacrococcygeal mass before the surgery

A male infant was born at 35.1 weeks with an SCT of 200 mm. The combined weight of the baby and teratoma was 4030 g (Fig. 6). His Apgar score was 9-10-10.

**Fig. 7:**
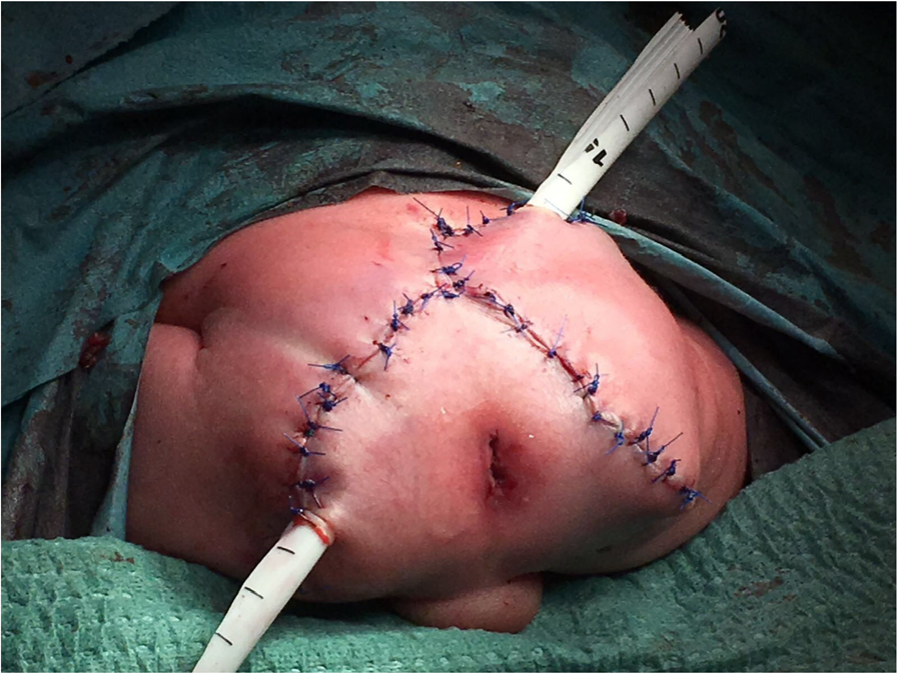
The result after surgery

Excision of the teratoma was performed at 36 hours of life, after embolization of the middle sacral artery. Surgery was done without incident, with an operative time of 3 hours. The reconstruction was done without any excess skin (Fig. 7). Pathological findings revealed an immature teratoma and no evidence of yolk sac tumor or other malignant elements. The postoperative alpha-fetoprotein (AFP) levels decreased quickly, being 150,000 before surgery and 64,500 afterward. The neonatal AFP values were followed during the first months with values of 14,915 at the 14th day after birth, 4136 at 6 months after birth, and 1.3 at 12 months of life. Other blood test parameters (including liver function, blood cell count, and hemostasis) were normal in both mother and infant samples.

The baby was discharged at 25 days after birth with normal results of abdominal, cerebral, and kidney ultrasound scans. Neonatal follow-up was performed during the first 16 months, and no long-term neurological deficits have appeared.

## Discussion

We present a case of SCT that was correctly diagnosed at the second-term scan and controlled by serial ultrasound examinations with a preterm termination that concluded with a successful multidisciplinary approach leading to good maternal and infant outcomes.

SCTs are an extragonadal neoplasm presenting in the presacral area as a mass in the midline caudal end of the fetus, and they can have solid, cystic, or mixed components. In most cases, they can be asymptomatic or cause rectal or bladder obstruction due to the growth of the tumor. Although more frequently reported in female infants, the malignant degeneration affects male infants in most cases [6, 7].

Histologically, SCTs can be classified as mature teratomas (fully differentiated tissues such as bone, teeth, and hair), immature teratomas (embryonal components or incompletely differentiated structures, which confer upon the tumor a major risk of malignancy), and malignant teratomas, which contain one or more of the malignant GCTs (yolk sac tumor, choriocarcinoma, embryonal carcinoma, and others) [8–10].

SCTs are also classified according to the American Academy of Pediatrics Surgical into four types, depending on the external or internal components of the tumor. While type I is primarily external, type II is external with a significant intrapelvic component, type III is external with a pelvic mass, and type IV is completely internal. The prenatal detection is easier in types I and II tumors, whereas the malignancy is more prevalent in type IV tumors [11, 12].

Prenatal diagnosis is usually made in the second trimester by routine sonography, even though it is also possible to be detected in the first trimester. The usual finding is a mass near the distal spine, but it could also present as an erosion in the vertebral bone or a group of calcifications. Besides the mass, other structural abnormalities, such as hydronephrosis, rectal stenosis, or cardiomegaly, could be found as a consequence of the presence of the tumor [13].

Magnetic resonance imaging can be useful in determining the extension of the tumor, the compression of adjacent organs, and in differentiating this pathology from a distal neural tube defect (myelocystocele or myelomeningocele), which is the most important differential diagnosis. The main difference is the location of the mass effect, which is presacral in SCTs and posterior in neural tube defects [14].

Ultrasound evaluation is used not only for the diagnosis but also for the monitoring of the tumor and its complications during the whole pregnancy. This evaluation should help in detecting which tumors are at high risk of causing complications that could cause hydrops and, in the end, fetal demise. Serial ultrasound should be performed to evaluate the tumor size, the solid or cystic portion, and the amount of amniotic fluid, as well as the vascular flow using Doppler. High-risk tumors are the ones that are larger or with a rapid growth, tumors that are mostly solid, and tumors with high vascular flow, because they can create a vascular steal phenomenon that can cause cardiomegaly and hydropic changes [10]. In these kinds of tumors, performing fetal echocardiography is recommended to evaluate the cardiac state and predict the possible onset of hydrops.

Perinatal morbidity and mortality in fetuses affected with SCTs is still high, mostly due to the associated prematurity that is usually involved. More important perinatal complications are preterm labor, malignant invasion, tumor bleeding or rupture, obstruction of the amniotic fluid, and heart failure [15].

The main treatment of SCTs is the surgical resection of the tumor and the coccyx to prevent the recurrence. This surgery is undertaken postnatally in most cases, and it is only performed *in utero* as a temporary measure in specialized centers and very selected cases that have a tumor with high risk of developing hydrops and a gestational age earlier than 32 weeks of gestation [16]. Delivery by cesarean section at 36 weeks is recommended in fetuses with low-risk SCT [17].

Normally, for SCTs without malignant elements, complete surgical resection is sufficient, followed by 3 years of measuring hormone levels of AFP and beta-human chorionic gonadotropin [18]. In malignant cases, adjuvant chemotherapy with a platinum-based regimen is suggested. SCTs have a recurrence rate of about 4% [19].

In our patient’s case, the ultrasound follow-up allowed us to identify the growth of the mass, high Doppler flow, and severe polyhydramnios, and, in this way, it let us plan the delivery, which was scheduled together with the neonatal service and the pediatric surgeon. This multidisciplinary approach permitted a premature surgery and consequently excellent neonatal outcomes.

## Conclusions

As shown in our patient’s case, prenatal ultrasound examination is important in the diagnosis as well as the monitoring of the tumor in order to detect those that could present a high risk of perinatal complications such as heart failure and hydrops. This prenatal evaluation is important to making an accurate diagnosis, an appropriate treatment, and a multidisciplinary approach.

## Data Availability

Data sharing is not applicable to this article, because no datasets were generated or analyzed during the current study.

## Abbreviations

AFP: Alpha-fetoprotein
GCT: Germ cell tumor
SCT: Sacrococcygeal teratoma
